# Utilization of dental health care services in context of the HIV epidemic- a cross-sectional study of dental patients in the Sudan

**DOI:** 10.1186/1472-6831-9-30

**Published:** 2009-11-16

**Authors:** Elwalid Fadul Nasir, Anne Nordrehaug Åstrøm, Jamil David, Raouf Wahab Ali

**Affiliations:** 1 Faculty of dentistry, University of Science and Technology, Omdurman, Sudan; 2 Centre for International Health, University of Bergen, Årstadveien 21 Bergen, Norway; 3 Department of Clinical Dentistry, Faculty of Medicine and Odontology, University of Bergen, Årstadveien 17 Bergen, Norway

## Abstract

**Background:**

HIV infected patients should be expected in the Sudanese dental health care services with an increasing frequency. Dental care utilization in the context of the HIV epidemic is generally poorly understood. Focusing on Sudanese dental patients with reported unknown HIV status, this study assessed the extent to which Andersen's model in terms of predisposing (socio-demographics), enabling (knowledge, attitudes and perceived risk related to HIV) and need related factors (oral health status) predict dental care utilization. It was hypothesized that enabling factors would add to the explanation of dental care utilization beyond that of predisposing and need related factors.

**Methods:**

Dental patients were recruited from Khartoum Dental Teaching Hospital (KDTH) and University of Science and Technology (UST) during March-July 2008. A total of 1262 patients (mean age 30.7, 56.5% females and 61% from KDTH) were examined clinically (DMFT) and participated in an interview.

**Results:**

A total of 53.9% confirmed having attended a dental clinic for treatment at least once in the past 2 years. Logistic regression analysis revealed that predisposing factors; travelling inside Sudan (OR = 0.5) were associated with lower odds and females were associated with higher odds (OR = 2.0) for dental service utilization. Enabling factors; higher knowledge of HIV transmission (OR = 0.6) and higher HIV related experience (OR = 0.7) were associated with lower odds, whereas positive attitudes towards infected people and high perceived risk of contagion (OR = 1.3) were associated with higher odds for dental care utilization. Among need related factors dental caries experience was strongly associated with dental care utilization (OR = 4.8).

**Conclusion:**

Disparity in the history of dental care utilization goes beyond socio-demographic position and need for dental care. Public awareness of HIV infection control and confidence on the competence of dentists should be improved to minimize avoidance behaviour and help establish dental health care patterns in Sudan.

## Background

The number of dentists in the public sector in Sudan has increased from 244 to 512 in the period from 2003 to 2007 and the dentist population ratio in Khartoum state (1.7:100000) is the highest in the country [1]. Dental care utilization of the public in light of the Human Immunodeficiency Virus (HIV) epidemic is so far poorly understood. With the exception of the Sudan the HIV epidemics in the Middle East and North Africa is comparatively small [2]. This is particularly so in Sub-Saharan Africa where an estimated 22 million people were living with HIV and Acquired Immune Deficiency Syndrome (AIDS) towards the end of 2007 and where access to dental health care services is commonly very limited [3,4]. In Sudan, the largest country on the Sub Saharan African continent, the prevalence of HIV and AIDS is still low. According to a population based study conducted in 2002, the sero-prevalence was estimated to be 1.6% [5,6]. However, being bordered by nine countries, some having a high prevalence of HIV and AIDS and having experienced long term ethnical and political conflicts, Sudan is vulnerable for an increase in the prevalence of HIV infections [5]. Thus, HIV infected patients should continue to be expected in the Sudanese dental health care services with an increasing frequency.

Oral lesions might be encountered at an early stage of HIV infection [7]. Weinert et al. [8] identified 16 oral conditions that might occur in HIV infected patients, seven of which can be suppressed by drug therapy. Oral health professionals can contribute to early diagnosis, prevention and treatment of HIV and AIDS infection [9]. Thus, it is recommended that HIV infected individuals should see a dentist regularly [10]. A number of studies have indicated unwillingness on the part of dental professionals to treat persons with HIV and AIDS due to fear of loosing non-HIV infected patients [11,12]. Since cross-infection might take place from patient to patient, from dentist to patient and vice versa, the advent of the HIV pandemic with increased awareness of cross infection among dental professionals and the public, has necessitated introduction of strict HIV protective procedures in dentistry [13,14]. However, poor compliance with standard infection control procedures have been reported, for instance in the South African Demographic and Health Survey of 1988 as well as more recent studies [15-17].

Recent findings based on the 1998 Community Health Assessment Project (CHAP) and the Behavioural Risk Factor Surveillance System (BRFSS) revealed that socio-demographics in terms of race (whites more likely to visit the dentist), income (higher income most likely to visit the dentist), education (higher education more likely to visit the dentist) and marital status (married most likely to visit the dentist) are the most important determinants of dental visiting habits in the general US population [14,18,19]. Several other factors have been reported to be associated with use of dental care, such as gender, non-poverty status, having a positive attitude towards dental health- and dental health care, having pain and being dentate [14,19,20]. Little is known, with respect to the public's HIV related knowledge, attitudes and fear of contagion in the dental environment and how such perceptions impact dental attendance patterns. Humphris et al. [21] reported that one third of the UK regular dental attendees believed that there was at least a slight risk of contracting HIV infection at the dental clinic. Lancaster et al [22] reported common misunderstandings regarding the public's knowledge about HIV and AIDS. In a Nigerian study of public perceptions of cross-infection control in dentistry, more than half of the respondents investigated felt that they could contract an infection in the dental clinic and 43% identified HIV as a risk [23]. Pistorius et al [24] examined dental patients in Germany and found that about 17% were generally afraid of contracting an infection at a dental office. Thomson et al [25] examined perceptions of cross infection in dentistry among Australians and found that 3.6% reported delayed or avoided dental visits due to perceived cross infection, the avoidance rate being highest in females and those who reported concern about cross infection control. A Mexican study revealed that only 21.2% of the study participants intended to continue treatment at a dental practice where patients with HIV were treated and 20% had similar intentions if the dentist was HIV positive [12]. To date, there has been no study exploring dental care utilization in the context of public knowledge of HIV and AIDS and perceived risk of contagion in the Sudan.

**Figure 1:**
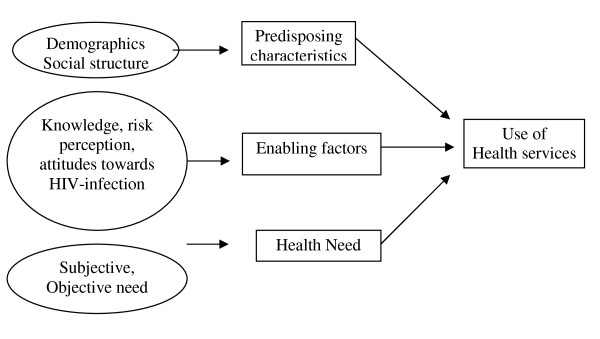
**Modified health service use behavioural model**.

This study applies Andersen's Behavioural model of Health services uptake (Figure 1) to guide the selection of variables to be associated with the utilization of dental health care services in Sudan [26]. According to this model, people's use of health service over a given period is a function of predisposing factors, enabling resources and treatment needs. Predisposing factors are based on the proposition that socio-demographics such as age, gender and ethnicity influence an individual's propensity to use health care services. Enabling factors include economic- and social resources that facilitate or impede use of health care whereas need related characteristics refer to the presence of clinically assessed and self-perceived disease status. Various predisposing and enabling factors might alter the use of health care services and the model suggests feedback loops, indicating that outcomes (*e.g*. use of health care services) might affect subsequent predisposing, enabling- and need related characteristics of individuals.

Focusing on a sample of Sudanese dental patients with reported unknown HIV status, this study assessed the extent to which the components of Andersen's model in terms of predisposing factors (socio-demographics), enabling factors (knowledge, attitudes and perceived risk related to HIV and AIDS infection in dental practices) and need related factors (clinically and self perceived oral health status) predict dental care utilization in terms of their relative contribution. It was hypothesized that enabling factors in terms of HIV related knowledge, attitudes and fear of contagion would add to the explanation of dental care utilization independent of predisposing- and need related factors.

## Methods

### Study participants

The present cross-sectional study was carried out from March to July 2008. Survey participants were recruited from dental clinics at two teaching hospitals in Khartoum state; Khartoum Dental Teaching Hospital (KDTH) and University of Science and Technology (UST). In both hospitals, all patients coming with dental complains are registered and then seen at the outpatients 'diagnostic' department for oral examination. All patients between 20 and 60 years of age with reported unknown HIV and AIDS status were invited to participate in the study. A total of 769 patients in KDTH (response rate 769/2650, 29.0%) and 491 patients in UST (response rate 491/950, 52%) consented to participate in a clinical examination, and an interview. Pre-test and post-test counselling was arranged before the conduction of the study. Reason for not participating was mainly due to time constraints on the part of patients and eagerness to receive the dental treatment. A sample size of 1200 patients was assumed to be satisfactory for a two-sided test assuming the proportion of dental care utilization in the previous 2 years to be 0.15 and 0.20 in patients with respectively low- and high education, a significance level of 5% and a power of 95%. Ethical permission was obtained from the Norwegian Regional Ethical Committee, Sudan National AIDS Programme (SNAP) and from the UST, and KDTH prior to conduction of the study.

### Oral Examination

One trained and calibrated dentist (EFN) conducted all clinical examinations in dental clinic settings equipped with an adjustable dental chair and artificial lightening. Examination was conducted using disposable gloves, sterilized dental mirrors, periodontal probes and dental explorers. Dental caries was recorded using the Decayed, Missing, Filled Teeth (DMFT) index, according to the guidelines by WHO [27] and recorded 0 or 1 (no caries experience, DMFT>0). Duplicate clinical caries registrations with 2 months interval were carried out among fourteen chair side dental assistants at UST. Intra examiner reliability in terms of Cohen's kappa for the DMFT components was 1.

### Interviews

A structured face-to-face interview including, questions on socio-demographic characteristics, oral health related behaviours, sources of HIV and AIDS related information, HIV related knowledge and attitudes was constructed in English and translated into Arabic by a dentist and then re-translated back to English by another dentist to check for consistency in the language. Two dentists (a male and a female) were assigned and trained for carrying out the interviews. Patients were interviewed in a confidential atmosphere while waiting for the clinical examination. The behavioural model proposed by Andersen was applied to identify variables to be considered relative to the use of dental health care services.

### Independent variables

*Predisposing factors *were assessed in terms of Socio-demographic factors; age, gender, hospital attended, profession, level of education and travelling inside/outside Sudan (Table 1). *Enabling factors *were assessed in terms of 1) amount of information about HIV and AIDS received from various sources, 2) knowledge on HIV transmission, 3) knowledge on HIV risk groups, 4) previous experience with HIV/AIDS, 5) perceived personal risk of contracting HIV AIDS, 6) attitudes towards people with HIV and AIDS, 7) attitudes towards HIV dental clinics. *Amount of information about HIV and AIDS from various sources *were assessed using 4 questions "How much information about HIV-related issues have you received from1) *radio/TV, 2) reading materials, 3) friends/relative*, and *4) health care workers*". Each question had response categories ranging from (1) "little" to (5) "very much". For cross tabulation each question was dichotomized into (0) "some/little received" (original categories 1, 2, 3) and (1) "much/very much information received" (original categories 4, 5). A formative sum score was constructed and dichotomized based on a median split yielding (1) "very much/much HIV information received" and (0) "some/little/none HIV information received". *Knowledge about modes of HIV transmission *was assessed using the statements: " HIV can be transmitted by 1) using contaminated sharp instruments, 2)unsafe blood transfusion, 3) shaking hands, 4) eating with infected people". Each statement had response category in the range from (1) "strongly disagree" to (5) "strongly disagree" for statements 1 and 2, and (1) "strongly agree" to (5) "strongly disagree" for statements 3 and 4. Each statement was dichotomized yielding (1) "correct knowledge" (original categories 4, 5) and (0) "incorrect knowledge" (original categories 1, 2, 3). A sum score of knowledge on transmission was constructed from the 4 dummy variables and dichotomized based on a median split into (1) "Correct overall knowledge" and (0) "Incorrect overall knowledge". *Knowledge of risk groups *was assessed by 5 statements with responses (1) "correct" and (0) "incorrect". The patients were asked "Is it correct or incorrect that university students, barbers, prisoners, soldiers, and health care workers are considered as high risk groups by profession"? One formative sum score was constructed from the 5 dummy variables. The sum score was dichotomized based on a median split into (1) "Correct knowledge on occupational risk groups" and (0) "Incorrect knowledge on occupational risk groups". *Previous experience with HIV and AIDS *was assessed using 3 items in terms of 1) "Have you known personally a person that is HIV positive? 2) Have you known personally any person that is sick with AIDS? 3) Have you known any person who has died because of AIDS?" Response categories were (1) Yes and (0) No. A sum variable was constructed from the three dummy variables and dichotomized based on a median split into 0 for "no experience" and 1 for "experience". *Perceived personal risk of contracting HIV and AIDS *was assessed by one question "How do you rate your own risk as a patient of contracting HIV *and *AIDS when attending dental practice. Responses were given on a scale in the range (1) No risk to (4) Great risk and dichotomized into (0) low risk (original categories 1, 2) and (1) high risk (original categories 3, 4). *Attitudes towards people with HIV and AIDS were assessed by *four statements; 1) I would go and visit a friend/relative if I knew that he/she had HIV *and *AIDS, 2) I would continue to be a friend with someone who got HIV-infection, 3) if a member of my family became sick with HIV *and *AIDS I would want this to remain secret, and 4) I would be willing to take care of someone with HIV *and *AIDS. Responses were given on 5-points Likert scales (1) strongly disagree to (5) strongly agree. Dummy variables (0) negative attitude, (1) positive attitude were constructed and added into a sum score. The sum score was dichotomized based on a median split into (0) for negative attitude towards people with HIV *and *AIDS, (1) for positive attitude towards people with HIV *and *AIDS'. *Attitudes towards dental clinics *were assessed by three statements; 1) *and *AIDS should be allowed to attend regular dental practices, 2) if a dentist/medical doctor has HIV but is not sick they should be allowed to continue their clinical work, 3) I would continue to visit a dental clinic if I knew that and AIDS were treated there. Responses were given on 5 point Likert scale (1) 'strongly disagree to (5) strongly agree. Dummy variables were constructed and added into a sum score. This sum score was dichotomized based on a median split into (0) for negative attitude towards dental clinics and (1) for positive attitude towards dental clinics. *Need related factors *were assessed in terms of "How do you perceive your general health status"?, "How do you consider the present condition of your mouth and teeth with categories (1) good, (0) bad and "Are you satisfied with the appearance of your teeth with categories (1) satisfied, (0) dissatisfied.

**Table 1:** Percentage distribution (n) of dental patients' socio-demographic characteristics (predisposing factors) in UST and KDTH hospitals:

Characteristic	UST % (n)	KDTH % (n)	Total % (n)
**Gender**			
Male	35.2 (173)	48.6 (373)	43.5 (548)
Female	64.8 (318)**	51.4 (394)	56.5 (712)
**Age**			
≤ 29	47.6 (233)	58.3 (447)	54.2 (682)
≥ 30	52.4 (257) **	41.7 (320)	45.8 (577)
**Marital status**			
Single	43.0 (211)	48.0(369)	54.2 (682)
In relationship	57.0 (280)	52.0 (400)	45.8 (577)
**Education**			
Primary/secondary	48.3 (237)	45.4 (349)	46.5 (586)
University and higher	51.7 (254)	54.6 (420)	53.5 (674)
**Profession**			
Unemployed, other	62.0 (304)	57.6 (442)	59.3 (746)
Technical, office, skilled labour	38.0 (186)	42.4 (325)	40.7 (513)
**Travelling inside Sudan**			
Yes	85.9 (420)	83.3 (639)	83.4 (1061)
No	14.1 (69)	16.7 (128)	15.7 (197)
**Travelling outside Sudan**			
Yes	41.5 (203)**	26.9 (206)	32.6 (410)
No	58.5 (286)	73.1 (561)	67.4 (848)

** p < 0.001, *p < 0.05

### Dependent variable

*Use of dental services *was assessed by asking "During the past 2 years - have you attended a dental clinic in order to receive treatment? Responses were given as (1) yes and (2) no.

### Statistical Methods

Data were analyzed using the Statistical Package for Social Sciences version 15.0 (SPSS Inc., Chicago, Illinois, USA). Bivariate analyses were conducted using cross-tabulations and Chi-square statistics. Determinants of use of dental care services were examined by multiple binary logistic regression analysis using the logistic model and 95% Confidence Interval (CI) whilst taking into account the hierarchical relationship between the various independent variables, as hypothesized by Anderson's model [28]. After controlling for predisposing factors (socio-demographics) at step I, enabling factors and need related factors were entered in step II and step III, respectively. Initially, multiple logistic regression analyses were conducted with the variables at each step separately (including all variables that were statistically significantly associated with utilization of dental care in bivariate analysis). Variables to be included in the various steps of the final hierarchical model were selected if p < 0.05 after adjustment for all other "same step" variables.

## Results

### Sample profile- predisposing factors by hospital of attendance

A total of 1262 dental patients participated in the study (mean age 30.7, Standard Deviation (SD) 8.5), 56.5% females and 61.0% from KDTH. Most of the participants (42.9%) resided in Omdurman city, followed by (31.4%) in Khartoum city, (16.2%) in Khartoum North city, and (9.5%) in other states. Table 1 gives the percentage distribution of participants' socio-demographic characteristics (predisposing factors) according to hospital of attendance. The patients attending UST were less frequently males (35.2% versus 48.6%), less frequently in the younger age group (47.6% and 58.3%), and had more frequently travelled outside Sudan (41.5% versus 26.9%) as compared to their KDTH counterparts.

### Enabling- and need related factors by hospital of attendance

Table 2 depicts the percentage distribution of sum scores of enabling (information received about HIV-related issues, knowledge on modes of transmission, knowledge on risk groups, personal experience of HIV and AIDS, perceived risk of HIV infection and attitudes towards dental clinics and HIV infected people) and need related factors (DMFT, perceived general and oral health) based on hospital of attendance. Moderate proportions of patients scored high on information received (39.5%), knowledge on transmission (73.3%) and knowledge on risk groups (66.8%). Moderate proportions had positive attitudes towards HIV dental clinics (49.6%) and people with HIV and AIDS (35.1%). A majority reported no experience with HIV infected people (75.6%), fear of HIV contagion in the dental environment (75.6%) and good oral (56.3%) and general health condition (73.3%). Compared with their UST counterparts, patients from KDTH were more frequently satisfied with teeth condition (50.2% versus 40.8%, p < 0.01), perceived less frequently a good general health (71.1% versus 76.8%, p < 0.05), and more frequently good teeth condition (61.0% versus 49.0%, p < 0.01). Compared with their UST counterparts, KDTH patients had more frequently positive attitudes towards dental clinics (39.0% versus 29.2%, p < 0.01), but showed less frequently positive attitudes towards HIV infected people (46.7% versus 54.1%, p < 0.05). The mean DMFT was 7.23, (SD 4.6) and the proportion of dental caries experience (DMFT>0) was 97.6%. The corresponding proportions of subjects with DT>0, FT>0 and MT>0 were respectively, 96.2%, 15.4% and 66.3%.

**Table 2:** Frequency distribution of enabling- and need related factors by hospital of attendance

	UST %(n)	KDTH % (n)	Total% (n)
**Enabling factors**			

**Information received**			
Low	57.7 (282)	62.3 (478)	60.5 (760)
High	42.3 (207)	37.7 (289)	39.5 (496)
**Knowledge on transmission**			
Low	72.3 (355)	74.6 (572)	73.3 (927)
High	27.7 (136)	25.4 (195)	26.3 (331)
**Knowledge on risk groups**			
Low	66.0 (305)	67.3 (432)	66.8 (737)
High	34.0 (157)	32.7 (210)	33.2 (367)
**Previous experience with HIV/AIDS**			
Yes	27.1 (133)	22.8 (175)	24.5 (308)
No	72.9 (358)	77.2 (592)	75.6 (952)
**Perceived personal risk as dental patients**			
Low	39.7 (195)	42.4 (325)	41.3 (308)
High	60.3 (296)	57.6 (442)	75.6 (952)
**Attitudes towards people with HIV-infection**			
Negative	45.9 (225)	53.3 (409)	50.4 (634)
Positive	54.1 (265)*	46.7 (358)	49.6 (624)
**Attitudes towards dental clinics**			
Negative	70.8 (347)	61.0 (468)	64.8 (815)
Positive	29.2 (143)	39.0 (299)**	35.1 (442)
**Need related factors**			
**DMFT**			
Zero	3.1 (15)	2.0 (15)	2.4 (30)
One or more	96.9 (476)	98.0 (754)	97.6 (1230)
**Perceived general health**			
Bad	23.2 (113)	28.9 (222)*	26.7 (335)
Good	76.8 (375)	71.1 (545)	73.3 (920)
**Perceived teeth condition**			
Bad	51.0 249)**	39.0 (299)	43.7 (548)
Good	49.0 (239)	61.0 (468)	56.3 (707)
**Satisfaction with teeth condition**			
No	59.2(289)**	49.8 (382)	53.5 (671)
Yes	40.8 (199)	50.2 (385)	46.5 (584)

** p < 0.001, *p < 0.05

### Predisposing-, enabling and need related factors associated with use of dental services during the past 2 years

A total of 53.9% of the patients, 61.7% of the UST and 49% of the KDTH patients, confirmed dental treatment at least once during the 2 years preceding the study. Of those who confirmed dental attendance, 77.1% reported attendance at least twice during that period. Table 3 and Table 4 depict the frequency distribution of dental care utilization by predisposing- enabling- and need related factors. Use of dental service varied systematically between male and females (43.7% versus 61.7%, p < 0.01) and between unemployed and employed patients (56.5% versus 49.9%, p < 0.05). Patients having travelled inside and outside Sudan reported dental visiting more frequently than those who had not travelled. Use of dental care varied with knowledge of HIV transmission, (55.8% versus 48.5% in patients with low and high knowledge), previous experience with HIV and AIDS (64.7% versus 50.4% in patients with less and more experience), perceived personal risk as a dental patient (49.3% versus 57.1% in patients with low and high risk perceptions) and attitudes towards HIV infected persons (48.3% versus 59.4% in patients with negative and positive attitudes). Use of dental care varied systematically with caries experience (20.0% versus 54.7% in patients without and with dental caries).

**Table 3:** Percentage distribution (n) of use of dental service by socio-demographic characteristics (predisposing factors).

Predisposing factors	Use of dental service % (n)
**Hospital of examination**	
UST	61.7 (301)**
KDTH	49.0 (376)
**Gender**	
Male	43.7 (239)
Female	61.7 (438)**
**Age**	
15-29	52.0 (354)
More than 30	56.2 (323)
**Marital status**	
Single	52.0 (354)
In relationship	56.2 (323)
**Education**	
Primary and secondary	53.4 (313)
University and higher	54.2 (363)
**Profession**	
Unemployed, other	56.5 (421)*
Technical, office, skilled labour	49.9 (255)
**Travelling inside Sudan**	
Yes	55.1 (583)*
No	46.9 (92)
**Travelling outside Sudan**	
Yes	55.1 (583)*
No	46.9 (92)

Percentages of those who confirmed dental care utilization during the 2 years preceding the study.

** p < 0.001, * p < 0.05

**Table 4:** Percentage distribution of use of dental care by enabling- and need related factors.

Enabling factors	Use of dental service % (n)
**Information received**	
Low	53.5 (406)
High	54.2 (269)
**Knowledge on transmission**	
Low	55.8 (516)*
High	48.5 (161)
**Knowledge on risk groups**	
Low	55.6 (410)
High	56.3 (206)
**Previous experience**	
No	64.7 (198)**
Yes	50.4 (479)
**Perceived risk of health workers**	
Low	50.9 (327)
High	57.0 (350)*
**Perceived personal risk as dental patients**	
Low	49.3 (257)
High	57.1 (420)
**Attitudes towards HIV-infected persons**	
Negative	48.3 (307)
Positive	59.4 (369)**
**Attitudes towards dental clinics HCW/patients HIV-infected**	
Negative	56.4 (459)*
Positive	49.1 (217)
**Need related factors**	
**DMFT**	
Zero	20.0 (6)
One or more	54.7 (671)**
**Perceived general health**	
Bad	45.1 (151)
Good	57.0 (526)**
**Teeth condition**	
Bad	65.9 (361)**
Good	44.6 (316)
**Satisfaction with oral condition**	
No	55.7 (374)
Yes	51.8 (303)

Percentages of those who confirmed dental care utilization during the 2 years preceding the study.

P value < 0.05 = *, P value < 0.01 = **

Initial unconditional regression analysis with dental care utilization as the dependent variable selected hospital attended, gender, travelling inside Sudan, travelling outside Sudan, knowledge of HIV transmission, perceived personal risk or HIV contagion, experience with patients with HIV, attitudes towards HIV infected patients, caries experience, perceived oral health status and perceived general health status to be included in the final logistic regression analysis. Predisposing factors in terms of hospital of attendance, gender, travelling inside/outside Sudan were entered in the first step explaining 6.9% of the variance in use of dental care (Nagelkerke's R square 0.069, p < 0.01). Entering enabling factors in step II in terms of knowledge on HIV transmission, personal experience with HIV/AIDS infected people, perceived risk of contagion in dentistry and attitudes towards HIV infected people raised the explained variance to 10.4% (Nagelkerke's R squared 0.104, p < 0.000). Entering need related variables in step III in terms of DMFT status, perceived oral health and general health raised the explained variance to a final 16.8% (Nagelkerke's R square 0.168, p < 0.01) (Table 5). In the final model, KDTH patients and patients that had not travelled inside Sudan were less likely whereas females were more likely to have attended dental care for treatment than were their counterparts in the opposite groups. Patients having high knowledge on transmission and high HIV-related experiences were less likely- and patients having positive attitudes and perceived high risk of HIV contagion were more likely to have used dental health care services than their counterparts in the opposite groups. Patients with caries experience and those who perceived good general health condition were more likely-, whereas patients who perceived good teeth condition were less likely to have attended dental care for treatment (Table 5). Stratified analyses revealed that the model explained 18% and 16% of the variance in dental care service use in KDTH and UST respectively. Statistically significant two way interaction was identified between perceived health- and DMFT status upon use of dental care. Stratified analyses by perceived health status revealed that among patients who perceived their health status to be good, those with caries were more likely than their caries-free counterparts to have visited a dentist during the 2 years preceding the study (OR = 10.3 95% CI 2.3-45.3). Among patient with bad health perceptions the relationship between DMFT status and dental health care services use was not statistically significant (OR = 1.8, 95% CI 0.5-6.3).

**Table 5:** Use of dental care regressed upon predisposing, enabling and need related factors: Odds ratios (OR) and 95% Confidence Intervals (95% CI)

	Adjusted OR (95% CI)	Adjusted OR (95% CI)	Adjusted OR (95% CI)
**Step 1 Predisposing factors**	Total sample	KDTH	UST
Travelling outside Sudan			
Yes	1	1	1
No	0.8 (0.6-1.0)	0.8 (0.6-1.2)	0.7 (0.4-1.1)
Travelling inside Sudan:			
Yes	**1**	**1**	1
No	**0.5 (0.4-0.8)**	**0.5 (0.3-0.8)**	0.6 (0.3-1.2)
Gender:			
Male	**1**	**1**	**1**
Female	**2.0 (1.5-2.4)**	**1.9 (1.3-2.6)**	**2.1 (1.4-3.2)**
Hospital:			
UST	**1**		
KDTH	**0.8 (0.5-0.9)**		
**Nagelkerke's R^2 ^= 0.069**			
**Step II Enabling factors**			
Knowledge HIV transmission:			
Low	1	1	1
High	**0.6 (0.4-0.8)**	**0.7 (0.5-1.1)**	**0.5 (0.3-0.7)**
Perceived personal risk:			
Low	**1**	1	1
High	**1.3 (1.1-1.6)**	1.2 (0.8-1.6)	1.3 (0.9-2.0)
Experience HIV/AIDS:			
Low	**1**	**1**	**1**
High	**0.7 (0.5-0.8)**	**0.6 (0.4-0.9)**	**0.8 (0.5-1.3)**
Attitudes towards HIV			
infected people:			
Negative	**1**	1	1
Positive	**1.3 (1.0-1.6)**	1.3 (0.9-1.7)	1.2 (0.8-1.8)
**Nagelkerke's R^2 ^= 0.104**			
**Step III Need related factors**			
DMFT: 0	**1**	1	**1**
DMFT>0	**4.8 (1.9-12.4)**	2.1 (0.6-7.1)	**14.9 (3.1-72.1)**
Teeth condition:			
Bad	**1**	**1**	**1**
Good	**0.5 (0.3-0.5)**	**0.4 (0.2-0.5)**	**0.5 (0.3-0.8)**
Health condition:			
Bad	**1**	**1**	**1**
Good	**1.9 (1.4-2.5)**	**2.7 (1.8-3.9)**	**0.9 (0.5-1.5)**
**Nagelkerke's R^2 ^= 0.168**			

* Controlled for age

## Discussion

This study is the first to confirm disparities in dental care utilization of Sudanese adult dental patients according to selected predisposing-, enabling- and need related factors. In accordance with the propositions of Andersen's behavioural model the present results confirmed the relationships between dental care utilization and socio-demographics (predisposing factors), HIV related knowledge, attitudes and perceived risk (enabling factors) and clinical (objective) - and subjective oral health indicators (need related factors). This suggests that use of dental health care services might be explained by variables organized into the three conceptual domains of predisposing, enabling and need-related factors among Sudanese adults attending university referral hospitals in Khartoum state. In addition, enabling factors, such as knowledge of HIV transmission, perceived personal risk of contagion, experience with HIV-infected people and attitudes towards HIV infected people contributed independently to the explained variance in dental care beyond that of predisposing- and need related factors. Nevertheless, the model explained only 18% and 16% of the variance in dental care utilization in KDTH and UST, indicating the importance of other influencing factors not accounted for in the present study, such as culture, dental cost, affordability and aspects of the Sudanese dental care system itself. Predisposing and need-related factors were the strongest predictors of dental care utilization in the total sample, as well as separately in the samples from UST and KDTH. Notably, it is not possible to assert that the present results demonstrate the crude impacts of the various factors considered since each could be biased by background confounding factors. Moreover, the study participants were patients attending two referral university hospitals for treatment, implicating that individuals who attended dental care for prophylactic- or other reasons, were excluded from the study group. This might have led to an overestimation of previous utilization rate since dental attendees that were excluded might also be low frequent users of dental care. They might also possess HIV-related attitudes and perceptions that are different from those of the respondents in the present study. Although this study provides valuable information by focusing on dental patients, it is unsure how close an approximation the present estimates are to the real situation of the general adult population in Khartoum state. Thus, studies based on random samples from the broader population could be recommended for future studies to provide answers to questions such as whether there are differences among dental attendees and non attendees regarding their perceptions of HIV-related issues and contagion in the dental environment.

About half of the investigated patients, and UST patients more frequently than KDTH patients, reported having received dental care at least once during the 2 years preceding the study. Evidently, the percentage of regular dental attendees varies across different populations. It has been reported to vary between 24% and 26% among adults in Tanzania and Nigeria, respectively [19,29,30]. In spite of the relatively high rate of dental attendance as identified by the present study, about 96% presented with untreated dental caries and half the sample was dissatisfied with their tooth condition (Table 2). Andersen's health behaviour model proposes that efficient access to health care services might be established when the level of health status improves relative to the amount of health care services received [26]. The present results indicating high levels of untreated dental caries irrespective of dental attendance frequency might point towards an inefficient access to oral health care services in Sudan. The number of dentists in the public sector in Sudan, has increased from 244 to 512 in the period from 2003 to 2007, which meant the expansion of coverage as well as access to dental services [1]. As shown in Table 4, higher rates of dental care utilization were found among patients having dental caries and perceiving a bad dental condition but also among those who were satisfied with their general health status, suggesting that the utilization pattern of health care services for medical and dental problems differ [29]. Thus, the burden from a bad general health condition might compete with contemporaneous effects of oral health problems regarding dental care utilization. This reasoning was supported by the identification of an interaction effect of perceived health condition and dental caries, indicating that dental caries did not impact utilization rates among participants considering their health condition to be bad. If a bad general health condition has higher priority compared to oral health needs, members of the public that are in bad physical condition will not receive appropriate levels of dental care. This accords with previous results in terms of people reporting more unmet need for dental health care than for general health care services [31].

Having been travelling inside Sudan and being a female increased the odds of having received dental care during the 2 years preceding this study. To the extent that travelling reflects higher socio-economic position of the participants, the present results accord with numerous studies globally showing that use of dental care occurs more frequently in females and socio-economically advantaged- compared with males and socio-economically disadvantaged groups [19]. The finding that females were the most frequent previous users of dental care is consistent with their reported propensity to possess more health related knowledge than men and also to be less likely to engage in health deteriorating behaviours. Unexpectedly, unemployed participants were more likely to use dental care than were their employed counterparts (Table 3). This result might reflect a "healthy worker effect" and also the fact that unemployed have more time to visit dental clinics than the employed part of the population. This finding is consistent with what has been reported in previous studies [10].

Although the majority of the participants were of higher education, most of them confirmed having received little HIV information, confirmed low levels of knowledge on transmission modes and risk groups, were un-experienced with HIV infected people and feared HIV contagion in the dental environment (Table 2). Whereas almost half of the study participants reported positive attitude towards HIV infected people, only a minority were in favour of ordinary dental clinics treating HIV infected patients. The relatively high level of fear of HIV infection observed among the study participants (75%) might reflect a certain scepticism about infectious control procedures taken by the dentist although being able to see infection control measures in action is not identical to either interpreting those measures or being aware their effectiveness. Studies from South Africa identified lack of protective eye wearing during dental procedures, not washing hands between patients, not disassembling an item prior disinfection/sterilization and not using sterile drill for each patient [17]. Studies of Nigerian patients and patients from industrialized countries have revealed that dental patients expect adequate infection control procedures and are informed that such measures are beneficial to both dental staff and patients [23]. On the other hand, large proportions of dental patients being totally ignorant to the sterilization methods utilized in dentistry have also been reported [32]. In light of previous studies suggesting that fear of contracting HIV tend to decrease with increasing level of education [24], the present results suggest that social resources related to HIV and AIDS seem to be influenced by factors other than people's educational level.

Being knowledgeable about modes of HIV transmission and having frequently been exposed to HIV and AIDS infected people seems to have impacted negatively- or acted as barriers towards dental care utilization (Table 4). On the other hand, having positive attitudes towards HIV infected people facilitated dental attendance. This is consistent with recent findings among dental attendees in Nigeria where about 60% of the study participants were unwilling to attend a dental clinic if they knew that patients with HIV were treated there [23]. Consistently, a German study of dental patients revealed that about 10% were in favour of separate waiting rooms for HIV infected patients [24]. An unwillingness to attend dental care on the part of patients being knowledgeable about transmission modes and experienced regarding HIV related issues is consistent with the findings of previous studies. Robinson and Croucher [33] investigated asymptomatic patients with HIV and found that among those who had attended dental care previously, 51% stopped after testing positive for HIV and AIDS. Personal experience with HIV and AIDS acting as a barrier towards utilization of dental care might be attributed to the fact that the estimated prevalence of HIV and AIDS is still low in Sudan and with a general trend of keeping HIV infection in secret [33]. Concern about HIV contagion in dental practice was associated with more frequent use of dental care in the multivariate analyses which seems at first counterintuitive but are consistent with findings among dental attendees in the United States [34]. In contrast, studies from other industrialized countries have shown that concern about HIV contagion in dental practices increases the likelihood of dental avoidance behaviour [25]. It is possible that among those who expressed increased concern about HIV contagion, anticipated impacts of not receiving dental care have outweighed any perceived risk of cross-infection. Considering the cross-sectional design of the present study, increased concern about HIV contagion in dental practice might be a consequence of frequent experience with dental care and with the application of improper cross-infection control procedures in the dental environments.

## Conclusion

The present data have shown that the components of Andersen's behavioural model explained 18% and 16% of the variance in dental care utilization of UST and KDTH patients, respectively. Enabling factors contributed independently to the explained variance in dental care beyond that of predisposing- and need related factors. Being knowledgeable about modes of HIV transmission and having positive attitudes towards HIV infected people impacted negatively and positively on dental care utilization. This suggests that disparities in dental care utilization of dental patients goes beyond socio-demographic position and need for dental care. According to the present study, dental patients had received little HIV information, confirmed low levels of knowledge on transmission modes and feared HIV contagion in the dental environment, whereas only a minority were in favour of ordinary dental clinics treating HIV infected patients. Together the present findings point to an urgent need for dental professionals and the government to address these disparities by improving public awareness of successful HIV infection control and confidence placed on the competences of dentists in order to minimize avoidance behaviour and to help establish dental health care patterns in this region.

## Competing interests

The authors declare that they have no competing interests.

## Authors' contributions

EFN: principle investigator, conceived of the study, designed the study, collected data, performed statistical analyses and manuscript writing. ANÅ: main supervisor guided the design of the study and has been actively involved in statistical analyses of data and in manuscript writing. JD: co-supervisor has been actively involved in statistical analyses and paper writing. RWA: co-supervisor has provided valuable comments on the paper in general and has been actively involved in manuscript writing. All authors read and approved the final manuscript.

## Pre-publication history

The pre-publication history for this paper can be accessed here:

http://www.biomedcentral.com/1472-6831/9/30/prepub

## References

[B1] National Health Information Centre http://fmoh.gov.sd/yearlyReports/2007%20report.pdf

[B2] UNGASS UNGASSoHA United Nations General Assembly Special Session on HIV/AIDS (UNGASS) Report 2006 - 2007

[B3] Kwan J Hospital formularies restrict evidence based practice BMJ 2005 3 331 515 10.1136/bmj.331.7515.515-b PMC1199068 16141168

[B4] Kwan SLY Petersen PE Pine CM Borutta A Health Promoting Schools: an opportunity for oral health promotion Bull World Health Organ 2005 83 677 685 16211159 PMC2626337

[B5] UNAIDS, UNICEF, WHOassessment of the epidemiological situation 2004 UNAIDS

[B6] UNAIDS Report on the global AIDS epidemic UNAIDS/0825E/JC1510E 2008 UNAIDS

[B7] MacPhail LA Greenspan D Feigal DW Lennette ET Greenspan JS Recurrent aphtous ulcers in association with HIV infection: Description of oral ulcer types and analyses of T-lymphocyte subsets Oral Surg Oral Med Pathol 1991 71 678 683 10.1016/0030-4220(91)90273-F 1676501

[B8] Weinert M Grimes RM Lynch DP Oral manifestations of HIV infection Ann Intern med 1996 125 485 496 8779462 10.7326/0003-4819-125-6-199609150-00010

[B9] Guest Editorial Strengthening the prevention of HIV/AIDS-related oral diseases: a global approach Community Dent Oral Epidemiol 2004 32 6 399 401 10.1111/j.1600-0528.2004.00192.x 15541154

[B10] Coulter ID Marcus M Freed JR Der-Martirosian C Cunnighan WE Andersen RM Maas WR Garcia I Schneider DA Genovese B Use of dental care by HIV infected medical patients J Dent Res 2000 79 356 361 10.1177/00220345000790060201 10890713

[B11] Bukar A Gofwen R Adeleke OA Taiwo OO Danfillo IS Jalo PH Discriminatory attitudes towards/AIDS by dental professionals in Nigeria Odontostomatol Trop 2008 31 34 40 19007095

[B12] Irigoyen-Camacho ME Zepeda-Zepeda MA Maupome G Lopez-Camara V Attitudes of a group of Mexico City residents toward HIV/AIDS in the dental office Am J Infect Control 2003 31 4 231 236 10.1067/mic.2003.30 12806361

[B13] DePaola D Howell H Baker CG Boy-Lefevre ML Hull P Holmstrup P Jerolimov V Hardwick K Lamster IB Lopez NJ Research and the dental student Eur J Dent Educ 2002 6 Suppl 3 45 51 10.1034/j.1600-0579.6.s3.6.x 12390258

[B14] Kohn WG Collins AS Cleveland JL Harte JA Eklund KJ Malvitz DM Guidelines for Infection Control in Dental Health-Care Settings, 2003 MMWR 2003 52 RR-17 1 61 14685139

[B15] Darling M Arendorf T Samaranayake LP Oral care of HIV-infected patients: the knowledge and attitudes of South African dentists J Dent Assoc S Afr 1992 47 9 399 402 9511621

[B16] Smith A Dickson M Aitken J Bagg J Contaminated dental instruments J Hosp Infect 2002 51 233 235 10.1053/jhin.2002.1213 12144804

[B17] Mehtar S Shisana O Mosala T Dunbar R Infection control practices in public dental care services: findings from one South African province J Hosp Infect 2007 66 65 10.1016/j.jhin.2007.02.008 17433494

[B18] Donaldson AN Everitt B Newton T Steele J Sheriff M Bower E The effects of social class and dental attendance on oral health J Dent Res 2008 87 1 60 64 10.1177/154405910808700110 18096895

[B19] Seirawan H Parsimonious prediction model for the prevalence of dental visits Community Dent Oral Epidemiol 2008 36 1 401 408 10.1111/j.1600-0528.2007.00420.x 18924256

[B20] Okullo I Åstrøm AN Haugejorden O Social inequalities in oral health and use of oral health care services among adolescents in Uganda Int J Paediatr Dent 2004 14 326 335 10.1111/j.1365-263X.2004.00568.x 15330998

[B21] Humphris GM Morrison T Horne L Perception of risk of HIV infection from regular attenders to an industrial dental service Br Dental J 1993 22 371 378 10.1038/sj.bdj.4808173 8494667

[B22] Lancaster DM Barsley RE Boozer CH Lundgren GA Public knowledge about AIDS: a survey of dental school patients. Part I Oral Surg Oral Med Oral Pathol 1991 71 3 377 385 10.1016/0030-4220(91)90322-4 2011369

[B23] Sofola OO Uti OG Onigbinde O Public perception of cross-infection control in dentistry in Nigeria Int Dent J 2005 55 383 387 16379143 10.1111/j.1875-595x.2005.tb00051.x

[B24] Pistorius A Willershausen B Heffner N Treatment aspects of patients with infectious diseases in dental practice-results of a survey Eur J Med Res 2002 7 10 457 462 12435625

[B25] Thomson WM Stewart JF Carter KD Spencer AJ Public perceptions of cross-infection control in dentistry Aus Dent J 1997 42 291 296 10.1111/j.1834-7819.1997.tb00132.x 9409043

[B26] Andersen RM National Health Surveys and the Behavioural Model of Health Services use Med Care 2008 47 647 653 10.1097/MLR.0b013e31817a835d 18580382

[B27] WHO Oral Health Surveys Basic Methods 1997 fourth Geneva: WHO

[B28] Andersen RM Revisiting the Behavioural Model and Access to Medical Care: Does It Matter? J Health Soc Behav 1995 36 1 10 10.2307/2137284 7738325

[B29] Sarita PT Tuominen R Use of health care services in two rural communities in Tanzania Community Dent Oral Epidemiol 1993 21 133 135 10.1111/j.1600-0528.1993.tb00737.x 8348785

[B30] Okunseri C Born D Chattopadhyay A Self-reported visits among adults in Benin City, Nigeria Int Dent J 2004 54 6 450 456 15633502 10.1111/j.1875-595x.2004.tb00303.x

[B31] Dobalian A Andersen RM Stein JS Hays RD Cunningham WE Marcus M The impact of HIV on oral health and subsequent use of dental services J Public Health Dent 2003 62 2 78 85 10.1111/j.1752-7325.2003.tb03479.x 12816137

[B32] Samaranayake LP McDonald KC Patient perception of cross-infection prevention in dentistry Oral Surg Oral Med Oral Pathol 1990 69 4 457 460 10.1016/0030-4220(90)90379-7 2326037

[B33] Robinson PG Croucher R Access to dental-care-experiences of men with HIV infection in the United Kingdom Community Dent Oral Epidemiol 1993 21 5 306 308 10.1111/j.1600-0528.1993.tb00780.x 8222607

[B34] Gerbert B Maguire BT Spitzer S Patients' attitudes towards dentistry and AIDS J Am Dent Assoc 1989 Suppl 16S 21S 2592688 10.14219/jada.archive.1989.0278

